# On the Road to Accurate Protein Biomarkers in Prostate Cancer Diagnosis and Prognosis: Current Status and Future Advances

**DOI:** 10.3390/ijms222413537

**Published:** 2021-12-17

**Authors:** Yiwu Yan, Su Yeon Yeon, Chen Qian, Sungyong You, Wei Yang

**Affiliations:** 1 Department of Surgery, Cedars-Sinai Medical Center, Los Angeles, CA 90048, USA; yiwu.yan@cshs.org (Y.Y.); suyeon.yeon@cshs.org (S.Y.Y.); chen.qian@cshs.org (C.Q.); sungyong.you@cshs.org (S.Y.); 2 Department of Biomedical Sciences, Cedars-Sinai Medical Center, Los Angeles, CA 90048, USA; 3 Samuel Oschin Comprehensive Cancer Institute, Cedars-Sinai Medical Center, Los Angeles, CA 90048, USA; 4 Department of Medicine, University of California Los Angeles, Los Angeles, CA 90095, USA

**Keywords:** prostate cancer, biomarker, proteomics, diagnosis, prognosis

## Abstract

Prostate cancer (PC) is a leading cause of morbidity and mortality among men worldwide. Molecular biomarkers work in conjunction with existing clinicopathologic tools to help physicians decide who to biopsy, re-biopsy, treat, or re-treat. The past decade has witnessed the commercialization of multiple PC protein biomarkers with improved performance, remarkable progress in proteomic technologies for global discovery and targeted validation of novel protein biomarkers from clinical specimens, and the emergence of novel, promising PC protein biomarkers. In this review, we summarize these advances and discuss the challenges and potential solutions for identifying and validating clinically useful protein biomarkers in PC diagnosis and prognosis. The identification of multi-protein biomarkers with high sensitivity and specificity, as well as their integration with clinicopathologic parameters, imaging, and other molecular biomarkers, bodes well for optimal personalized management of PC patients.

## 1. Introduction

Prostate cancer (PC) is a leading cause of morbidity and mortality in men, particularly in developed countries, resulting in enormous social and economic costs. PC is the second most commonly diagnosed non-skin cancer and the fifth most lethal cancer in males worldwide. It was estimated that around 1.4 million men will be diagnosed with PC in 2020, with approximately 375,000 PC patients dying from this disease [1]. The incidence and mortality rates of PC are strongly associated with age—the median ages at diagnosis and death are 67 and 80, respectively (https://seer.cancer.gov/statfacts/html/prost.html (accessed on 20 November 2021)). Global aging is expected to result in around 16% of the global population being over 65 by 2050, up from 9% in 2019 [2]. As a result, the prevalence of PC and its economic cost are expected to skyrocket in the coming years.

Currently, the cornerstones of PC management include serum prostate-specific antigen (PSA) quantification, digital rectal examination (DRE), and systemic transrectal ultrasound (TRUS)-guided biopsies. PC is frequently discovered before symptoms emerge due to the widespread use of PSA and DRE screening. If the screening results are abnormal, approximately 12 needle core biopsies (small pieces of tissue) are collected from various locations of the prostate for histological analysis. To diagnose PC, a pathologist examines the collected biopsy tissue under a microscope for aberrant histological alterations.

PC is a remarkably heterogeneous disease that can range from indolent to very aggressive [3]. It can be divided into a number of intermediate clinical states, each of which may benefit from a different therapeutic modality. As a result, if a pathologist detects cancer, the next step is to determine the PC’s aggressiveness so that the patient can receive optimal care. Gleason grading, developed by Donald F. Gleason in 1966 [4], is widely regarded as the best predictor of prognosis in localized PC. The Gleason grading system categorizes PC tumors into five Gleason patterns, ranging from well differentiated (grade 1) to poorly differentiated (grade 5) [3]. Because PC tumors often contain cancer cells of varying grades, each PC tumor is assigned two Gleason grades (the most prominent and the second most prominent), the sum of which is reported as a Gleason score (GS) ranging from 2 to 10. However, in contemporary PC diagnosis, GSs of 2–5 are rare, so they are almost exclusively 6–10. To reflect the current diagnostic and therapeutic approaches, the GS system was modified to categorize PCs into five distinct Grade Groups (GGs) [5]. These are GG1 through GG5, which correspond to GSs of ≤6, 7 (3 + 4), 7 (4 + 3), 8, and 9–10, respectively [3].

Despite its utility, the Gleason grading does not accurately predict disease outcomes for individual patients and is subject to inter-observer variability. To further improve risk stratification, various tools were developed by combining GS with other clinicopathologic parameters, such as PSA, tumor-node-metastasis (TNM) classification, age, and percentage of positive biopsies [3]. Among these risk assessment tools, the most widely used ones include the Partin table, the D’Amico risk group, the Cancer of the Prostate Risk Assessment (CAPRA) score, and the Kattan nomogram [3]. However, the performance of these tools in predicting PC aggressiveness is suboptimal. For example, some PCs classified as intermediate-risk tumors are actually high risk. Thus, additional information is required to better assess the risk of a given patient.

## 2. Clinical Needs of Molecular Biomarkers in PC Diagnosis and Prognosis

**Figure 1:**
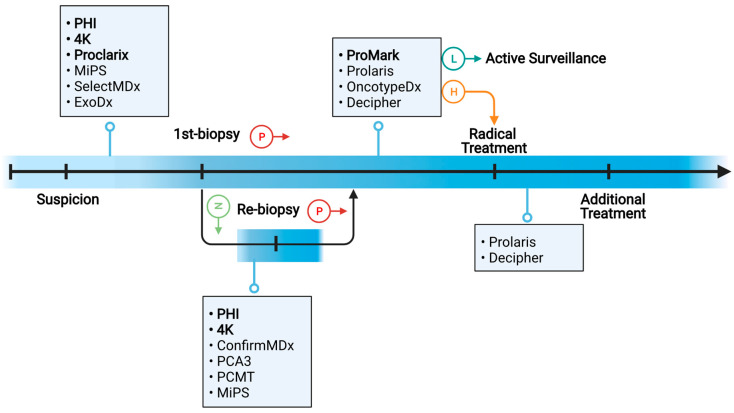
Schematic overview of the clinical needs for molecular biomarkers in various settings. Boxes present commercially available PC biomarkers, among which protein biomarkers are in bold font.

Molecular biomarkers supplement existing clinicopathologic tools for PC diagnosis and prognosis by providing additional and valuable information about the biological behavior of PC tumors. To improve the management of PC patients, a number of molecular biomarkers have been developed to address the following questions (Figure 1).

First, who should be biopsied? When PC tumors are diagnosed early, they can be treated with surgery and radiotherapy, which can be curative. However, only about 30% of prostate biopsy procedures result in PC diagnosis [6,7,8]. Furthermore, approximately 40% of positive biopsies detect clinically insignificant (GS ≤ 6, i.e., GG = 1) PC [7], which is rarely fatal if untreated. Prostate biopsies are not without risks; they can cause patient anxiety and discomfort, as well as potential side effects such as pain, fever, blood in the urine, and infections [9]. Therefore, biomarkers are needed to detect clinically significant (GS ≥ 7, i.e., GG ≥ 2) PC while minimizing unnecessary and invasive prostate biopsies. Commercially available molecular biomarkers for this purpose include Prostate Health Index (PHI), four-kallikrein (4 K) score, Proclarix, Mi Prostate Score (MiPS), SelectMDX, and ExoDx [10,11].

Second, after a negative initial biopsy, who should be re-biopsied? Because PC is a multifocal disease, and only a small proportion (~1%) of the prostate is sampled, the standard biopsy strategy is prone to sampling error, missing 25–35% of all PC and 10–20% of clinically significant PC [12]. Therefore, if the initial systemic biopsy result is negative but clinical suspicion of PC persists (e.g., continued elevation of serum PSA levels), patients are recommended to be re-biopsied. Total serum PSA levels and serum PSA kinetics, as currently used, are ineffective indicators of PC and frequently result in unnecessary repeat biopsies [13]. To improve prediction accuracy, newer molecular biomarkers have been developed. Among these, commercially available biomarkers include PHI, 4 Kscore, ConfirmMDx, PCA3, Prostate Core Mitomix Test (PCMT), and MiPS [10,11].

Third, after being diagnosed with PC, should a patient receive definitive treatment or be monitored by active surveillance (AS)? In the hope of a cure, approximately 87% of patients with newly diagnosed PC elect for definitive treatments such as radical prostatectomy (RP) and radiotherapy (RT) [8]. However, such treatments can cause significant complications such as urinary, bowel, and sexual dysfunction, lowering the quality of life of PC patients. In comparison, AS allows PC patients to avoid the significant side effects of PC treatment until their disease progresses to the point where treatment is required (if at all). Thus, AS is increasingly being used on patients with low-risk or favorable-intermediate-risk PC [14]. To distinguish PC patients who can be safely monitored by AS from those who require definitive treatment, accurate risk stratification is the key. Because prostate biopsies only sample a small portion of the prostate gland, clinicopathological risk assessment on biopsy specimens is inherently flawed. According to a large-scale study involving 10,273 patients, 44% of low-risk cases were upgraded and 9.7% were up-staged at RP [15]. On the other hand, 18–62% downgrading could occur between needle biopsy and RP [16]. For higher-resolution risk stratification, a number of molecular biomarkers including ProMark, Prolaris, Oncotype DX, and Decipher have been developed and commercialized [10,11,17]. These tests assess underlying biology from biopsy tissue and thus partially address the issues of tumor heterogeneity and biopsy under-sampling.

Fourth, who needs additional treatment following radical PC treatment? A large-scale study found that at 10 years after RP, the cumulative incidence of biochemical recurrence (BCR) and metastasis was 13% and 6%, respectively, among 3089 men with intermediate- or high-risk PC defined by the National Comprehensive Cancer Network (NCCN) [18]. Nonetheless, risk stratification based on clinicopathologic risk factors is inadequate and should be improved. The aforementioned biomarkers such as Decipher and Prolaris have been used in the post-RP setting to improve the prediction of PC recurrence risk [19].

This review will focus on protein biomarkers in PC diagnosis and prognosis because of the following reasons. First, molecular biomarkers (particularly genomic biomarkers) in PC diagnosis and prognosis have already been extensively reviewed [10,11,20,21,22], yet few review articles specifically focus on PC protein biomarkers. Second, proteins are major functional molecules in cells and the primary determinants of most phenotypic traits. Hence, protein biomarkers have a high clinical potential, particularly for routine monitoring, because their expression can reflect disease activity in real time. Last, the past few years have witnessed remarkable progress in global or targeted protein quantification, allowing for the discovery and validation of novel protein biomarkers with clinical relevance. Because the Special Issue focuses on biomarkers for diagnosis and prognosis of urological tumors, we will not discuss protein biomarkers for treatment response or resistance. Here, we will first review Food and Drug Administration (FDA)-approved and commercially available protein biomarkers for PC diagnosis and prognosis. We will then discuss the biological sources for biomarker discovery and their pros and cons. Next, we will describe proteomic approaches for the global discovery and targeted validation of novel PC protein biomarkers. Last, we will discuss the challenges and potential solutions for identifying clinically useful PC protein biomarkers.

## 3. FDA-Approved and Commercially Available Protein Biomarkers for PC Diagnosis and Prognosis

PSA, a kallikrein-like serine protease glycoprotein encoded by the *KLK3* gene, was approved by the FDA in 1986 as a prognostic biomarker in PC and then in 1994 as a diagnostic tool for PC detection among asymptomatic men. It is the best validated and most widely used molecular PC biomarker employed by clinicians. However, PSA is prostate-specific but not PC-specific. It could be elevated not only in PC but also in non-cancerous conditions such as benign prostatic hyperplasia (BPH) and prostatitis. As a result, the positive predictive value (PPV) for a PSA level of >4.0 ng/mL is only about 30% [23]. In other words, slightly less than one in three men with an elevated PSA will have PC detected on biopsy. Furthermore, PSA has a low specificity for clinically significant (GG ≥ 2) PC, resulting in the over-diagnosis of clinically insignificant (GG = 1) PC. Consequently, a large number of men undergo unnecessary prostate biopsies. Additionally, PSA screening can produce false-negative results. A cutoff of 4.0 ng/mL is estimated to miss about 15% of PC cases, including 2.3% of clinically significant PC cases [24]. Due to the limitations of PSA, novel biomarkers for detecting clinically significant PC have been developed. Among these are several commercially available protein biomarkers, including serum-based PHI, 4 KScore, and Proclarix, as well as biopsy tissue-based ProMark.

PHI (Beckman Coulter) combines total PSA (tPSA), free PSA (fPSA), and [−2] proPSA (p2PSA) into a single score to predict the likelihood of PC on biopsy. tPSA comprises free (unbound) PSA and bound (predominantly to α-1-antichymotrypsin) PSA. fPSA is PSA unbound to any carrier molecules or proteins. p2PSA is a truncated form of proPSA (the inactive precursor of PSA) that contains two pro-leader amino acids. In a 2011 multicenter study of 892 patients, PHI demonstrated an AUC of 0.70 for PC in men with 2–10 ng/mL PSA and normal DRE, outperforming p2PSA (0.56), fPSA (0.62), and tPSA (0.53) [25]. PHI was premarket approved by the FDA in 2012 (No. P090026) for PC diagnosis in men over 50, with 4–10 ng/mL PSA and a negative DRE.

The 4 Kscore (OPKO Health) test measures the protein levels of four kallikreins—namely, tPSA, fPSA, intact PSA, and human kallikrein 2—and then combines these parameters with clinical information on age, DRE outcome, and prior negative biopsy status into an algorithm. It is reported as a percentage likelihood of harboring GG ≥ 2 PC (0–100%). In a large prospective validation study of 1012 men, 4 Kscore had an AUC of 0.82 for predicting GG ≥ 2 PC [26].

Proclarix (Proteomedix) combines tPSA and fPSA with cancer-related glycoproteins thrombospondin-1 (THBS1) and cathepsin D (CTSD) as well as age to calculate a patient’s probability of having clinically significant PC on biopsy [27]. It is intended for use in men with a prostate volume ≥ 35 mL, no history of PC, a normal DRE result, and elevated serum PSA levels (2–10 ng/mL). At a sensitivity of 90%, the Proclarix test has a specificity of 42%, a negative predictive value (NPV) of 95%, and a PPV of 25% [27].

ProMark (MetaMark Genetics) measures the expression levels of eight proteins that are involved in cell signaling, stress response, and cell proliferation [28]. These proteins are SMAD4, PDSS2, HSPA9, FIS, YBOX1, DERL1, and CUL2. ProMark is a personalized prognostic test to distinguish patients with early-stage PC (GS = 3 + 3 and 3 + 4) for AS from those who require RP. Using a quantitative multiplex immunofluorescence platform, the ProMark test measures the expression levels of the eight proteins in formalin-fixed biopsy tissue specimens. Subsequently, ProMark reports a score ranging from 0 to 1 that reflects the probability of detecting adverse prostate pathology in the same patient’s RP specimen [29]. For ProMark scores of > 0.8 or 0.9, the predictive value for unfavorable pathological characteristics after RP can be as high as 76.9% and 100%, respectively [29]. According to the latest NCCN guidelines (version 1.2022) [17], ProMark is recommended for men with very-low-risk or low-risk PC on biopsy, a life expectancy of at least 10 years, and no prior PC treatment.

## 4. Clinical Biospecimens for the Discovery and Validation of PC Protein Biomarkers

Despite their clinical utility, the aforementioned FDA-approved and commercially available PC protein biomarkers lack the specificity and sensitivity needed to confidently adjust the course of PC biopsy and treatment. Thus, novel PC protein biomarkers with improved diagnostic and prognostic values are urgently needed. Common clinical biospecimens for the discovery and validation of such biomarkers include patient tissue, expressed prostatic secretions (EPS), EPS-urine, serum/plasma, and extracellular vesicles (EVs) isolated from biofluids. The benefits and drawbacks of these clinical specimens will be discussed further below. We will not discuss preclinical models such as PC cell lines, patient-derived xenografts (PDXs), organoids, and transgenic or genetically engineered mouse models. Using these preclinical models can be a good starting point for directing biomarker discovery towards PC-relevant pathways and increasing confidence in existing biomarker candidates. Nonetheless, they fall short of fully capturing the vast heterogeneity of human PC.

Tissue is frequently used in the discovery of PC protein biomarkers because it closely reflects tumor biology. Compared with biofluid specimens, tissue specimens allow for more direct sampling of proteomic changes in tumor cells and the microenvironment. Nevertheless, prostate tissue biopsy is invasive and only provides a limited snapshot of the tumor. Furthermore, prostate biopsy under-samples multi-focal prostate tumors, making a comprehensive view of PC tumors in individual patients difficult.

Prostate-proximal fluids include direct-EPS and EPS-urine. Direct-EPS is a prostatic fluid collected from patients undergoing RP by massaging the organ and expelling 0.5–1 mL of fluid immediately prior to surgical removal. It is anticipated to contain a relatively high concentration and purity of prostate-secreted proteins. Despite this, EPS has limited clinical utility and cannot be obtained longitudinally because it is collected just prior to RP. EPS-urine, also called post-DRE urine, is first-catch urine collected after a DRE. EPS-urine contains a small fraction of EPS that is expelled during the DRE and collected by the urine. It is easy to obtain and can be collected longitudinally.

Serum/plasma-based biomarkers are particularly appealing in the context of PC because they can be routinely measured pre-, post-, or on-treatment and assayed alongside PSA. Serum/plasma-based liquid biopsies are more likely to fully capture diverse information that reflects intratumoral, microenvironmental, and systemic conditions than tissue biopsies, which only sample ~1% of the prostate. Furthermore, serum/plasma biomarker analysis can be performed in a fast and high-throughput manner, which is critical in a clinical setting to determine a more appropriate PC management plan in a timely fashion. Nonetheless, serum/plasma is currently not very suitable for biomarker discovery using mass spectrometry (MS). This is because tumor-derived proteins are present at low concentrations in the circulation, making them difficult to detect using MS-based shotgun proteomics. Furthermore, serum/plasma proteins have a dynamic range of 10–12 orders of magnitude (from <5 pg/mL to ~50 mg/mL), whereas MS only covers a dynamic range of 4–5 orders of magnitude [30]. As a result, most low-abundance serum/plasma proteins cannot be reliably detected and quantified by MS. Immunodepletion can be used before MS analysis to remove high-abundance proteins, making low-abundance proteins easier to detect and quantify. However, immunodepletion has some limitations such as variable depletion efficiencies for high-abundance proteins, concomitant loss of non-targeted proteins, and decreased sample preparation throughput [31].

EVs are phospholipid biolayer membrane-coated vesicles released by most cell types in physiological and pathological conditions [32]. Of note, EVs are more abundant in biofluid samples from PC patients than from control subjects [33]. EVs are highly heterogeneous in size, biogenesis, function, content, and membrane markers [34]. Because tumor-derived EVs carry tumor-specific cargos and are released into various human body fluids, EVs represent an attractive source of cancer biomarkers. Because EV contents are well protected within a lipid membrane, they are stable in circulation. To isolate EVs from biological fluids, a variety of techniques and commercial products have been developed. Common isolation methods include (1) ultracentrifugation, (2) precipitation, (3) ultrafiltration, (4) size-exclusion chromatography, (5) affinity interactions, and (6) microfluidic devices and microchips [35]. However, standardized methods for EV isolation remain to be established so that they can be transferred across research or clinical labs.

## 5. Proteomic Approaches for Global Discovery of Novel Protein Biomarkers from Clinical PC Specimens

### 5.1. MS-Based Protein Biomarker Discovery

MS is a biophysical technique that allows for the structural analysis of various biomolecules in the form of gas-phase ions, resulting in their detection and quantification. MS can be broadly classified into bottom-up and top-down MS. In bottom-up MS, proteins are digested by an endoproteinase (e.g., trypsin) into peptides prior to MS analysis. In comparison, top-down MS analyzes intact proteins. Compared with top-down MS, bottom-up MS is more well established and approximately two orders of magnitude more sensitive [36]. At present, bottom-up MS enables the identification and quantification of thousands of different proteins in cultured cells and tissue specimens. Thus, it has served as the workhorse tool for the global discovery of novel protein biomarkers.

**Figure 2:**
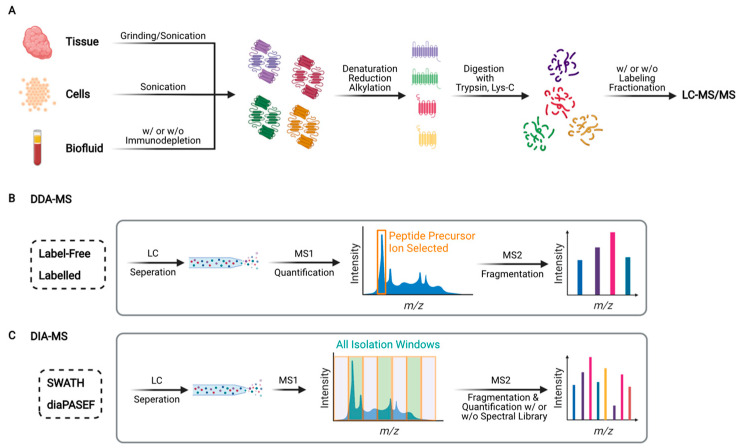
Schematic overview of bottom-up proteomics. (**A**) Typical workflow for global proteomic analysis. Proteins are extracted from tissues, cells, or biofluids, subsequently digested into peptides by an endoproteinase (e.g., trypsin or Lys-C), followed by label-free or isotope labeling-based quantitative proteomic analysis using liquid chromatography-tandem mass spectrometry (LC-MS/MS). (**B**) Schematic of data-dependent acquisition (DDA)-mass spectrometry (MS). Peptides are separated by reversed-phase LC and converted into positively charged gas-phase precursor ions, whose mass/charge (*m/z*) ratios are measured by MS. Peptide precursor ions with the highest intensities are isolated and broken into product (fragment) ions by MS/MS (also called MS2). (**C**) Schematic of data-independent acquisition (DIA)-MS. Representative methods include sequential window acquisition of all theoretic mass spectra (SWATH) and data-independent acquisition parallel accumulation-serial fragmentation (diaPASEF). Unlike DDA-MS, DIA-MS acquires MS/MS scans with wide isolation windows (e.g., 25 *m/z*) that do not target any particular peptide precursor ions.

In bottom-up (also known as shotgun) proteomics, extracted proteins are digested with MS-grade endoproteinases to obtain peptides prior to liquid chromatography (LC)-tandem mass spectrometry (MS/MS) analysis (Figure 2A). Trypsin and Lys-C are the most commonly used endoproteinases because they yield peptides with positively charged C-termini, which are amenable to ionization and thus detectable by MS. The sample complexity can be reduced further if peptides are fractionated using LC methods such as high-pH reversed-phase LC or strong cation exchange LC. Peptide fractionation improves proteomic coverage (i.e., number of detected proteins), but it requires substantially longer instrument time and more input material (typically >40 ug of protein).

In shotgun proteomics, two independent MS scan modes are commonly used: data-dependent acquisition (DDA) and data-independent acquisition (DIA) (Figure 2B,C). DDA-MS was first reported in the 1990s [37], and it has since become the standard for global proteomics [38]. DIA-MS was first described in the early 2000s [39,40]. However, it did not gain widespread acceptance until the introduction of sequential windowed acquisition of all theoretical fragment ion spectra (SWATH) in 2012 [41]. Later, other DIA-MS methods were developed, including multiplexed MS/MS [42] and data-independent acquisition parallel accumulation-serial fragmentation (diaPASEF) [43]. In DDA-MS, MS/MS scans are acquired with narrow isolation windows, e.g., two units of mass/charge (*m/z*) ratio, centered on peptide precursor ions with the highest intensities (typically top 10–20) in an MS scan. In DIA-MS, MS/MS scans are acquired with wide isolation windows (e.g., 25 units of *m/z* ratio) that do not target any particular peptide precursor ions [44]. Because DDA-MS stochastically selects precursor ions, whereas DIA-MS consistently collects fragment ion spectra for all detected precursor ions, the latter provides better reproducibility than the former [41]. Nonetheless, owing to continuous improvements in the speed and sensitivity of mass spectrometers, as well as the development of novel algorithms that enable global targeting of thousands of peptides (e.g., MaxQuant.Live [45]), the stochastic nature of DDA-MS is becoming less problematic. Moreover, DDA-MS can be coupled with stable isotope labeling to achieve highly multiplexed protein quantification [46]. For instance, TMTpro enables simultaneous quantification of proteins in up to 16 different samples in a single LC-MS/MS run [47,48]. In comparison, DIA-MS is predominantly label-free and samples are analyzed individually. Although several multiplexed DIA-MS methods, such as NeuCoDIA [49] and MdFDIA [50], have been developed, their utility in cancer biomarker discovery remains to be proven.

Notably, a major advantage of MS-based proteomics is that it can analyze not only protein abundance but also various post-translational modifications (PTMs) and protein–protein interactions (PPIs) on the proteome scale [38]. Widespread PTMs include protein phosphorylation, glycosylation, and palmitoylation. Drake et al. applied DDA-MS-based label-free tyrosine phosphoproteomics to compare 35 prostate tissue specimens, including 18 metastatic castration-resistant PC (mCRPC) and 12 primary PC samples [51]. The study identified 297 phosphotyrosine peptides and revealed intra-patient similarity and inter-patient heterogeneity of activated tyrosine kinases such as SRC, EGFR, RET, and ALK. Later, the same group performed global phosphoproteomics analysis of 34 tissue specimens including 16 mCRPC samples and 11 treatment-naive localized PC samples [52]. A total of 8051 phosphopeptides were identified, and six major signaling pathways were found to be enriched in mCRPC specimens when compared with other samples. Liu et al. conducted N-glycoproteomic analysis of PC tissue specimens, including 10 normal prostate, 24 non-aggressive PC, 16 aggressive PC, and 25 metastatic PC, by SWATH-MS [53]. They discovered and validated that two glycoproteins encoded by *NAAA* and *PTK7* were significantly associated with PC aggressiveness. Dong et al. performed a global N-glycoproteomic analysis of 74 aggressive PC and 68 non-aggressive PC urine samples [54]. The study showed that a three-protein signature including urinary ACPP, urinary CLU, and serum PSA provides an AUC of 0.86 in distinguishing aggressive PC from non-aggressive PC. Protein palmitoylation (more accurately known as *S*-acylation) is a widespread PTM that plays critical roles in reversibly regulating protein localization, activity, stability, and complex formation [55,56]. For global analysis of palmitoylated proteins, we developed Palmitoyl-Protein Identification and Site Characterization (PalmPISC) [57] and low-background Acyl-biotinyl Exchange (LB-ABE) [58]. Using these palmitoyl-proteomics approaches, we profiled the palmitoyl-proteomes in platelets [59] and EVs shed by PC cells [60], identifying the substrate proteins of DHHC3 in PC and breast cancer [61]. We are currently applying palmitoyl-proteomics to analyze total plasma samples and plasma EVs from PC patients, which holds great potential for uncovering novel PC protein biomarkers. Nonetheless, global analysis of protein PTMs to identify novel protein biomarkers remains limited, mainly due to the requirement for a relatively large amount of proteins (typically 500–1000 µg per sample) and a longer time for sample processing.

PPIs are frequently rewired in diseases including cancer [62,63]. Thus, aberrant PPIs are promising and likely more specific disease biomarkers than abnormal protein expression. We are the first to identify protein complexes associated with PC progression in clinical tissue specimens on the proteome scale [64]. In this proof-of-concept study, we coupled tandem mass tagging (TMT)-synchronous precursor selection (SPS)-MS/MS/MS (MS3) with differential expression and co-regulation analyses to compare the differences between protein complexes in PC-adjacent normal prostate, low-grade PC, and high-grade PC tissue specimens (*n* = 9 in each group). Our study identified 28 differentially assembled protein complexes in low-grade PC versus normal prostate, 22 differentially assembled protein complexes in high-grade PC versus normal prostate, and 22 differentially assembled protein complexes in high-grade PC versus low-grade PC. Further exploitation of these deregulated protein complexes is anticipated to reveal novel PPI biomarkers for aggressive PC.

Table 1 summarizes candidate protein biomarkers that have been identified by MS-based discovery proteomics. Well-known biomarkers such as KLK3/PSA, FOLH1/PSMA, and TMPRSS2 were repeatedly identified. Interestingly, TGM4 was the second most frequently identified after KLK3/PSA. TGM4 is upregulated in PC patients with higher Gleason scores and higher PSA levels, and its protein levels correlate with tumor recurrence [65].

Despite its many strengths, MS-based discovery proteomics has some drawbacks. First, it is biased toward high-abundance proteins and provides less robust detection and quantification of low-abundance proteins. Second, analyzing complex biological matrices such as serum and plasma remains a formidable challenge. Despite progress [85,86], it remains difficult to detect proteins below low µg/mL or high ng/mL levels without extensive sample fractionation or protein enrichment. Of note, serum/plasma protein biomarkers with clinical applicability are often present in the pg/mL to sub ng/mL range [30]. Third, MS instruments are expensive, and their operation requires specialized skills, limiting widespread adoption in clinical labs. These limitations can be addressed, at least in part, by immunoassay- or aptamer-based discovery proteomics as described below.

### 5.2. Immunoassay-Based Protein Biomarker Discovery

#### 5.2.1. Antibody Array

**Figure 3:**
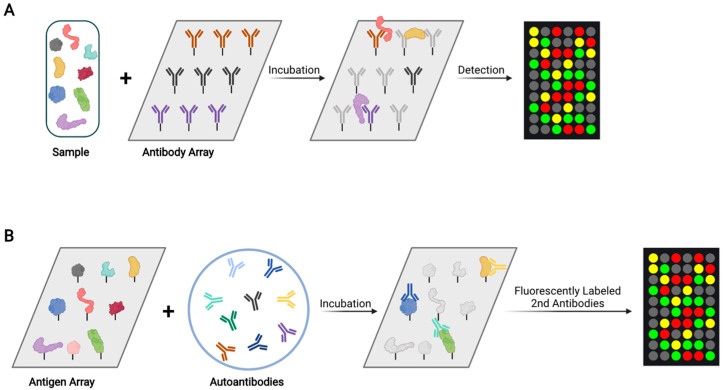
Schematic overview of antibody array and antigen array analyses. (**A**) In an antibody array, each spot contains one type of antibody and each array is incubated with one test sample. For protein quantification, proteins are fluorescently labeled (either directly or indirectly) and incubated with an antibody array. (**B**) In an antigen array, each spot contains one purified protein and each array is incubated with one test sample. For protein quantification, fluorescently labeled secondary antibodies are incubated with an antigen array.

In an antibody array, a large collection of distinct capture antibodies is immobilized onto a solid support surface (Figure 3A). For an assay, each antibody array is incubated with one test sample, where tens to hundreds of different proteins and phosphoproteins are measured simultaneously. For protein quantification, proteins in samples are labeled by one or more fluorescent dyes. The dye labeling can be direct via chemical conjugation. Alternatively, proteins can be biotinylated so that they can be probed with fluorescently labeled streptavidin [87]. Miller et al. used antibody arrays containing 184 different antibodies to analyze serum samples from 33 PC patients and 20 controls, leading to the identification of five differentially expressed proteins: von Willebrand factor, immunoglobulin M, α1-antichymotrypsin, villin, and immunoglobulin G [88]. Shafer et al. used antibody arrays containing 102 antibodies to analyze serum samples from healthy controls, organ-confined PC, non-organ-confined PC, and benign prostatic conditions (*n* = 92 in each group), resulting in the discovery of some differentially expressed proteins including thrombospondin 1 [89]. However, antibody arrays may suffer from the limitations of batch-to-batch variability, antibody stability, and high cost. As a result, antibody arrays have not yet been frequently used to identify novel PC biomarkers.

#### 5.2.2. Antigen Array

Tumor-associated autoantibodies are promising diagnostic and prognostic biomarkers [90]. They are easily accessible in blood specimens, have a long half-life, and may be significantly more abundant than tumor antigens due to antibody amplification response [90]. Each array contains purified proteins spotted onto nitrocellulose filters that are adhered to glass slides. For analysis, arrays are incubated with patient samples, and tumor-associated autoantibodies in the samples bind to their cognate antigens on the array (Figure 3B). After washing, the arrays are incubated with fluorescently labeled secondary antibodies. The fluorescence intensities of bound autoantibodies are used to quantify them. Using an array containing 123 tumor-associated antigens, Adeola et al. measured autoantibodies in serum samples from 20 PC patients, 32 BPH patients, and 15 controls [91]. They identified 41 candidate PC biomarkers, including GAGE1, ROPN1, SPANXA1, and PRKCZ.

#### 5.2.3. Proximity Extension Assay (PEA)

**Figure 4:**
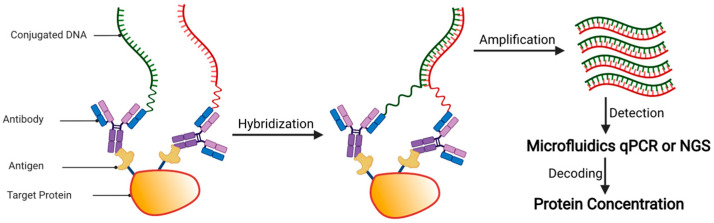
Schematic overview of proximity extension assay (PEA) analysis. Upon sample incubation, the antibody-based proximity probe pair binds to its specific antigens on the same protein. As a result, the pair of probes come in close proximity and hybridize. The addition of a DNA polymerase causes the hybridizing oligo to be extended, resulting in a DNA template that can be detected and quantified by quantitative PCR (qPCR) or next-generation sequencing (NGS).

The PEA technology (Olink Proteomics) was developed based on proximity-dependent DNA ligation (Figure 4) [92]. In PEA, either two matched monoclonal antibodies, or one batch of polyclonal antibody split into two fractions, are covalently linked with two different 40-mer oligonucleotides at the 3′- and 5′-end, respectively. To the 3′-linked probe, a 56-mer DNA oligo that consists of 40 nt complementary to that probe, 7 nt spacer, and 9 nt complementary to the corresponding 5′-linked probe is hybridized. The hybridized proximity probe pair is then incubated with a sample that contains the antigens of interest, resulting in antigen-proximity probe pair binding. As a result, the oligonucleotides come into contact with one another and hybridize. The hybridizing oligo is then extended over the other probe arm using a DNA polymerase. The resulting DNA template can be detected and quantified by quantitative polymerase chain reaction (qPCR) or next-generation sequencing (NGS).

Using the Olink Immuno-oncology and Oncology II panel, Liu et al. measured the abundance of 92 target proteins in serum samples from men without PC, patients with low-risk primary PC, patients with high-risk primary PC, and patients with metastatic PC (*n* = 20 per group) [93]. Nine proteins (i.e., PTN, MK, PVRL4, EPHA2, TFPI-2, hK11, SYND1, ANGPT2, and hK14) were found to be elevated in metastatic PC patients, compared with other groups. In another study, the same group applied PEA to measure the protein levels of 184 target proteins in pre- and post-operative serum samples from ten patients with high-grade and high-volume PC [94]. Six proteins were found to be significantly reduced after RP: CASP8, MSLN, FGFBP1, ICOSLG, TIE2, and S100A4.

Currently, the Olink Explore 1536/384 allows for the quantification of 1463 distinct proteins (https://www.olink.com/products-services/explore/ (accessed on 25 November 2021)). An expanded version of the platform, Olink Explore 3072, allows for the quantification of ~3000 unique proteins and is available for pre-order. It is expected that Olink will be increasingly applied to identify protein biomarkers for PC diagnosis and prognosis.

### 5.3. Aptamer-Based Protein Biomarker Discovery

Aptamers, also known as chemical antibodies, are short oligonucleotide sequences that have a high affinity for binding to a protein target. Aptamers have several advantages as follows [95]. First, they are not immunogenic, making them suitable for clinical use. Second, due to their chemical synthesis and modification, aptamers can be more easily, quickly, and economically produced than antibodies, resulting in little inter-batch variability. Third, aptamers are highly stable in harsh environments, ensuring longer shelf life and easier storage and transport. Fourth, because of their nanometric size, aptamers can interact with a wide range of targets, ranging from inorganic molecules to whole cells, as well as being able to penetrate tissues and internalize into cells. Fifth, aptamers can be screened without prior knowledge of the target molecules, allowing the discovery of previously unknown biomarkers.

**Figure 5:**
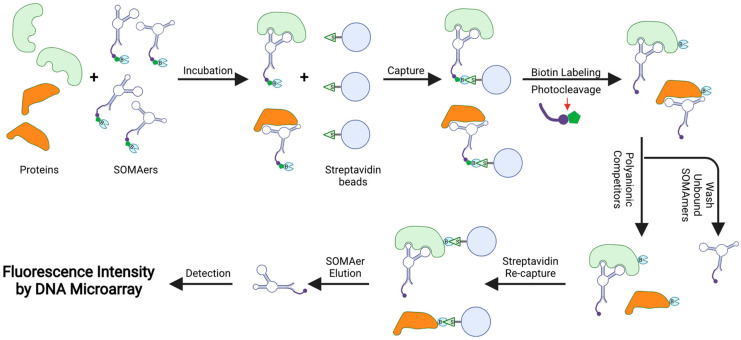
Schematic overview of the SOMAscan analysis. Each SOMAmer contains a biotin (B) group, a photo-cleavable link, and a fluorescent tag at the 5′ end. SOMAmers are mixed with the test sample, forming SOMAmer–protein complexes. The complexes are captured on streptavidin beads via strong biotin–streptavidin interaction. The captured proteins are then biotinylated and the SOMAmer–protein complexes are released from beads using ultraviolet light. Polyanionic competitors are added to promote the dissociation between proteins and non-specific SOMAmers. The SOMAmer–protein complexes are recaptured on new streptavidin beads. Protein-bound SOMAmers are eluted, hybridized to custom arrays of SOMAmer-complementary oligonucleotides, and quantified by fluorescence intensities, which are proportional to the concentrations of their cognate target proteins.

The slow off-rate modified aptamer (SOMAmer) assay (SOMAscan) is a multiplex proteomic platform (Figure 5). In SOMAscan, each SOMAmer is modified with a biotin group, a photocleavable group, and a fluorescent tag. After incubating SOMAmers with the sample, any formed SOMAmer–protein complexes are captured by streptavidin beads via streptavidin–biotin interaction. The captured proteins are then biotinylated, and the complexes are released from the beads via photocleavage and washing. Another set of streptavidin beads is then added to the mixture to recapture the SOMAmer–protein complexes via the biotinylated proteins. SOMAmers are eluted using specific pH conditions and hybridized to complementary DNA sequences on a proprietary microarray chip. The concentrations of SOMAmers, which are proportional to target protein concentrations, are quantified by fluorescence intensity.

To identify biomarkers indicative of treatment failure, Welton et al. applied SOMAscan to analyze EVs isolated from 11 plasma samples and 5 urine samples [96]. However, no proteins were found to be significantly different between the treatment-naïve and treatment-resistant groups. In another study, Dudani et al. used SOMAscan to compare five PC samples and five matched normal adjacent tissue samples [97]. Proteases such as uPA and PRSS3 were found to be more abundant in the PC samples than in the normal adjacent tissue samples.

The SOMAscan workflow is highly automated, allowing for high-throughput analysis of 7000 proteins in 680 samples in a single day (https://somalogic.com/life-sciences/ (accessed on 25 November 2021).). It was reported that the median inter-assay coefficient of variation (CV) was ~5% [98]. SOMAscan is expected to be used more frequently in discovering novel PC biomarkers, especially those in body fluids. However, it should be noted that about 7% of SOMAmers have cross-reactivity to another protein [99].

## 6. Computational Approaches for Prioritizing Protein Biomarker Candidates

**Figure 6:**
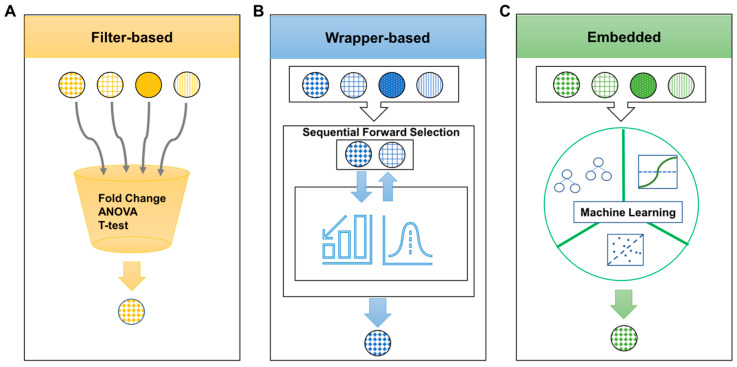
Schematic of three different feature selection methods for determining optimal protein biomarkers. (**A**) Filter-based methods are based on choosing the differential feature according to discriminating metrics such as *p*-value. Metrics are calculated from a statistical method such as fold change, ANOVA, and Student’s *t*-test. This method ranks proteins according to the selected criteria that put highly redundant or differentially expressed proteins on the top rank. (**B**) Wrapper-based methods look for the best subset of features based on their predictive power. Generation of a feature subset and assessment function is repeated until the optimal subset is returned through the learning algorithm. The feature subset with the highest performance is returned as a result. Sequential forward selection is one of the examples of this method and uses a bottom-up search technique to find the best subset. (**C**) Embedded methods use various machine learning techniques to select the optimal subset of features. Random forest, support vector machine and artificial neural network are examples of embedded feature selection methods.

A single biomarker can only provide a limited diagnostic or prognostic value. It is widely accepted that a panel of multiple biomarkers is more clinically useful than a single molecular biomarker. One key challenge is determining the best combination of individual biomarkers from massive omics data sets. For this, three feature selection methods are available: filter-based, wrapper-based, and embedded (Figure 6) [100]. Filter-based feature selection is computationally fast and simple, yet interaction with the classifier is ignored. Commonly used methods include fold change, ANOVA, Student’s *t*-test, and Mann–Whitney–Wilcoxon test (Figure 6A). Wrapper-based feature selection looks for the best subset of features based on their predictive power, but it is compute-intensive. Methods in this category include sequential forward selection, sequential backward elimination, and genetic algorithm (Figure 6B). Embedded feature selection may require a dataset to be randomly divided into training and testing sets to avoid model overfitting or underfitting. Methods for this include decision tree, support vector machine, and random forest (Figure 6C), all of which have been applied to identify optimal combinations of PC protein biomarkers [101,102,103].

## 7. Proteomic Approaches for Multiplexed Targeted Validation of Candidate PC Biomarker Proteins

Multiplex protein measurements reduce time, cost, and sample volume. Currently, commonly used multiplexed targeted proteomics methods include antibody-independent targeted MS as well as antibody-dependent reverse phase protein array (RPPA) and Luminex. These methods are complementary and should be integrated to achieve optimal results.

### 7.1. MS-Based Multiplexed Targeted Proteomics

**Figure 7:**
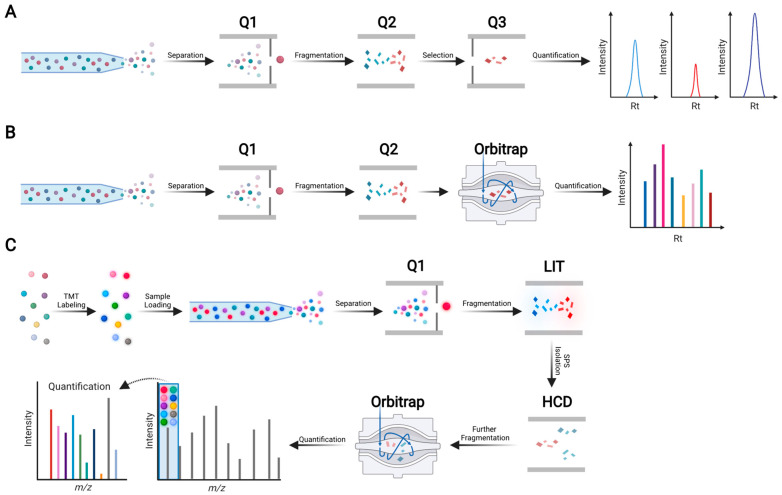
Schematic overview of MS-based targeted proteomics methods. (**A**) Schematic of selected reaction monitoring (SRM), also known as multiple reaction monitoring (MRM). For peptide quantification, three to five selected fragment ions from a single peptide precursor ion are measured sequentially. SRM is typically performed on a triple quadrupole (QqQ) mass spectrometer. The first quadrupole (Q1) isolates a predefined peptide precursor ion, the second quadrupole (Q2) is a collision cell where isolated precursor ions are broken into product ions (also called fragment ions), and the third quadrupole (Q3) isolates predefined product ions. Such predefined pairs of precursor and product ions are called transitions, which provide high specificity and sensitivity to quantify peptides that are surrogates of proteins of interest. (**B**) Schematic of parallel monitoring reaction (PRM). PRM employs a high-resolution Orbitrap mass analyzer to simultaneously monitor many product ions. Because transitions do not need to be defined in advance, PRM is easier to set up than SRM. (**C**) Schematic of TOMAHAQ (triggers by offset, multiplexed, accurate mass, high-resolution, absolute quantification). Peptides derived from 10 (or 16) samples are labeled with 10-plex (or 16-plex) tandem mass tag (TMT) reagents, which consist of 10 (or 16) different isobaric compounds with the same mass and chemical structure. Subsequently, an equal amount of differentially TMT-labeled peptides is pooled into one tube, followed by LC separation and targeted MS analysis. Rt: retention time; LIT: linear ion trap; HCD: higher-energy collisional dissociation.

Currently, the most widely used MS-based multiplexed targeted proteomics approaches are selected reaction monitoring (SRM) [104], also called multiple reaction monitoring (MRM), and parallel reaction monitoring (PRM) [105,106] (Figure 7A,B). SRM and PRM assays are typically carried out in triple quadrupole (QqQ) (e.g., QTRAP series) and quadrupole-orbitrap (Q-OT) (e.g., Q Exactive series) mass spectrometers, respectively. In SRM, a predefined series of transitions (i.e., precursor–product ion pairs) is monitored over time for precise quantification of each target peptide. In PRM, targeted MS/MS is applied to simultaneously monitor all product ions of a targeted peptide with high resolution and mass accuracy. Unlike SRM, the selection of the best transitions in PRM can be defined in a post-acquisition step. SRM and PRM have similar linearity, dynamic range, and precision, with the latter requiring less method development than the former [107,108]. Notably, once validated on an instrument, SRM or PRM assays can be transferred across sites and clinical laboratories. Table 2 summarizes SRM- or PRM-based targeted proteomics validation studies of candidate PC protein biomarkers.

SRM and PRM are both label-free targeted proteomics methods, and samples must be analyzed separately (1–2 h per sample). In comparison, TOMAHAQ (triggered by offset, multiplexed, accurate mass, high-resolution, absolute quantification) combines sample multiplexing with targeted proteomics to significantly increase throughput (Figure 7C) [111]. For instance, using TOMAHAQ, 131 different peptides were quantified across 180 cell lysate samples in only 48 h [111]. Nevertheless, TOMAHAQ can only be implemented on expensive tribrid mass spectrometers such as Orbitrap Eclipse and Orbitrap Fusion Lumos, limiting its widespread use in research and clinical applications.

### 7.2. Antibody-Based Multiplexed Targeted Proteomics

Compared with MS, antibody-based immunoassays provide higher throughput and sensitivity. For example, the enzyme-linked immunosorbent assay (ELISA) is one of the most widely used tools for protein quantification in research settings, as well as the gold standard in clinical laboratories for detecting single analytes. Notably, ELISAs can achieve 1–10 pg/mL detection limits without sample pretreatment [112]. Nevertheless, ELISA is not well suitable for multiplexed targeted proteomics because it requires a relatively high sample volume (as high as 50 µL sample per analyte) for analysis as well as high cost (USD 100,000—USD 1,000,000 per assay) and long lead time (1–2 years) for assay development [113]. To enable multiplexed protein detection and quantification, several immunoassay technologies, such as RPPA and microsphere bead capture, have been developed and commercialized [114].

#### 7.2.1. RPPA

**Figure 8:**
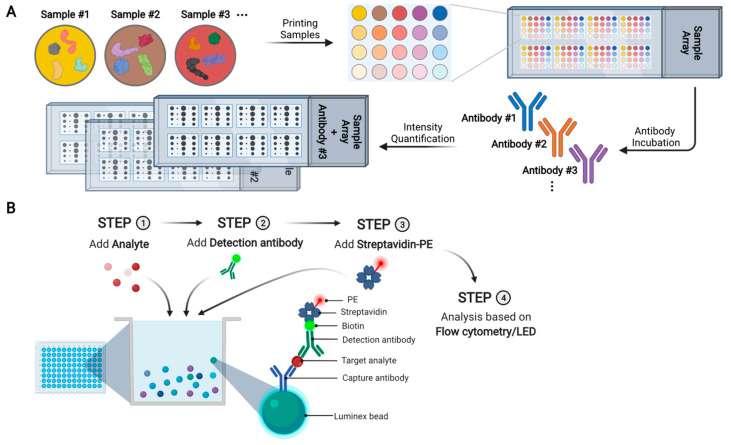
Schematic overview of reverse phase protein array (RPPA) and Luminex microsphere bead capture. (**A**) Schematic of RPPA. Each spot contains a single sample and each array is probed with one specific antibody. The bound antibody can be quantified (either directly or indirectly) by fluorescent, colorimetric, or chemiluminescent assays. (**B**) Schematic of Luminex microsphere bead capture assay. Analyte-specific capture antibodies are immobilized on superparamagnetic microsphere beads that are color-coded. After incubating a test sample with antibody-coated microsphere beads, target proteins are captured. Biotinylated detection antibodies specific to the target proteins are added, leading to the formation of an antibody–antigen sandwich. Phycoerythrin (PE)-conjugated streptavidin is added, so that the protein amount can be quantified based on the intensities of PE-derived signal.

RPPAs are widely used for high-throughput, multiplexed, and quantitative analysis of target proteins and phosphoproteins in tissue lysates, cultured cell lines, and, to a lesser extent, biological fluids (Figure 8A) [115]. Each array can contain hundreds of different test samples, one for each spot. Of note, owing to the high sensitivity of RPPA, only a very small amount of protein (equivalent to ~200 cells) is required for each test sample. Each array is also printed with control samples containing varying amounts of protein, allowing for the generation of a calibration curve for protein quantification. In RPPA analysis, each array is probed with one single antibody that can be detected using fluorescent, colorimetric, or chemiluminescent assays. The analytical sensitivity of RPPA has been reported to range from picogram to femtogram levels, with a CV of <15% [116].

The first time RPPA was used was in the study of PC [117]. In this study, PC progression was found to be significantly associated with increased AKT1 phosphorylation, decreased ERK phosphorylation, and suppression of apoptosis pathways. In another study, RPPA was used to examine signaling pathways in normal, tumor, and stromal cells isolated from PC tissue specimens using laser capture microdissection (LCM) [118]. AKT1 and GSK3β phosphorylation levels were higher in tumor cells than in normal cells, whereas ERK, p38, and PKCα phosphorylation levels were lower. RPPA has also been applied to analyze tumor cells isolated by LCM from treatment-naïve localized PC, hormone-refractory localized PC, and metastatic PC tissue specimens [119]. The study found that ERBB2 and BCL-2 phosphorylation levels were higher in metastatic PC than in primary PC, whereas ERK, p38, and JNK phosphorylation levels were lower. In yet another study, RPPAs were probed with 190 validated antibodies to analyze 152 primary PC samples [120]. The study identified three clusters with (1) high apoptosis and DNA damage response pathway activities, (2) a high EMT pathway score, and (3) increased PI3K–AKT, MAPK, and RTK activities, respectively. To explore the molecular architecture of the tumor microenvironment in human PC, RPPAs were probed with 124 antibodies to analyze epithelial and stromal cells isolated by LCM from 18 PC patients [121]. The study identified a protein network activated in the malignant PC tumor microenvironment. In the so-far largest RPPA analysis of PC specimens, RPPAs were probed with 225 validated antibodies to analyze 351 primary PC specimens and 7312 patient samples from 30 other cancer types [122]. The study showed that the level of PI3K–AKT–mTOR pathway activity in primary PC was average among the 31 evaluated cancer types. More recently, RPPA was used to measure key antigens and activated signaling in EVs isolated from PC patients’ sera [123]. The study showed that PD-L1, ERG, Integrin-β5, Survivin, TGF-β, phosphorylated TSC2, and partners of MAPK and mTOR pathways are differentially expressed in tumor-derived EVs.

Taken together, these studies demonstrate that RPPA is a valuable tool for targeted proteomics analysis of PC specimens, owing to its high throughput, sensitivity, cost-effectiveness, and quick turnaround time. Therefore, RPPAs are particularly useful in the clinical setting. It should be noted, however, that RPPA is antibody-dependent, necessitating extensive antibody and assay validation.

#### 7.2.2. Microsphere Bead Capture (Luminex)

Figure 8B shows a schematic of microsphere bead capture analysis. The analyte-specific capture antibodies are immobilized on 6.5 µm superparamagnetic microsphere beads that are color-coded. For multiplexed targeted proteomics, a mixture of antibody-coated beads is used to capture target proteins. Subsequently, biotinylated detection antibodies specific to the target proteins are added to form an antibody–antigen sandwich. Phycoerythrin (PE)-conjugated streptavidin is added to bind to the biotinylated detection antibodies. Beads are read on a dual-laser flow-based detection instrument: one laser classifies the beads, and the other laser determines the magnitude of the PE-derived signal that is proportional to the target protein bound to the beads. Experiments can be performed in 96- or 384-well microtiter plates, allowing for high throughput. The limit of detection is about 1–10 pg/mL, and the dynamic range is about 3–4 orders of magnitude [124]. In practice, up to about 30 target proteins can be analyzed in each assay [124].

Tsaur et al. used Luminex to measure the concentrations of six cytokines in serum samples from 39 PC patients and 15 healthy donors [125]. They found that CCL2 was significantly more abundant in the serum samples of PC patients compared with controls, suggesting that CCL2 is a potential diagnostic biomarker for PC. Al-Mazidi et al. used Luminex to analyze 27 cytokines in plasma samples from 19 healthy controls, 29 untreated patients with nonmetastatic PC, 20 patients with metastatic PC who received chemotherapy and reported pain, and 10 chemotherapy-treated patients with no pain [126]. They found that the concentrations of IL-6, IL-8, Eotaxin, VEGF, and IP-10 are significantly higher in the plasma of chemotherapy-treated patients with pain than the other groups. These cytokines are potential targets for pain control in PC patients receiving chemotherapy. Shore et al. applied Luminex to measure the concentrations of 32 cellular growth factors in serum samples from 64 patients with non-aggressive PC and 120 patients with aggressive PC [127]. The concentrations of PSA, IL-7, and VEGF were found to be significantly higher in aggressive PC than non-aggressive PC.

## 8. Challenges and Potential Solutions in Identifying and Validating Clinically Valuable PC Protein Biomarkers

In the past decade, a large number of candidate protein biomarkers for PC diagnosis and prognosis have been identified. Nonetheless, very few have been approved for clinical use, and their spread in the clinical routine is very slow. The reasons are multi-faceted, and we offer potential solutions as follows. First, most studies stop at the discovery phase and fail to proceed to biomarker verification and validation, largely due to a lack of financial support, patient specimens, or both. A small number of studies proceeded to biomarker verification, yet few have used a completely independent cohort of samples to rule out false positives caused by sample collection and processing. To address this issue, collaborative efforts involving multiple institutions and, more optimally, multiple nations are required for large-scale validation studies of prioritized biomarker candidates. Second, most discovery cohorts are small in size, and few studies share the same biomarker candidates due to a lack of standardized methods for sample collection and processing, data acquisition, and bioinformatics analysis. Given the wealth of available data on biomarker candidates, a meta-analysis would help prioritize biomarker candidates for large-scale validation. Third, rather than identifying protein biomarkers that can provide additional information to the established clinicopathological parameters (e.g., Gleason grading), many studies identified proteins that are proxies for these parameters. As a result, the clinical values of such candidate protein biomarkers may be insignificant. A potential solution is to identify candidate protein biomarkers associated with clinically relevant end-points, such as the occurrence of metastases, disease-specific mortality, and overall survival. Fourth, most PC protein biomarker candidates were identified using specimens from white men, so their clinical values for patients of other races (e.g., African American) remain unknown. To address this issue, biomarker discovery studies should be conducted in more diverse cohorts of patient specimens. Fifth, PC is a highly heterogeneous disease, so the discovery of protein biomarkers with both high sensitivity and high specificity is inherently difficult. To overcome this issue, a large sample size is required for biomarker discovery, and a relatively large panel of protein biomarkers is very likely required to recapitulate the heterogeneity. Sixth, it remains unclear how the short-term and long-term temporal dynamics of biomarkers affect their validity and clinical utility. Longitudinal studies are needed to address this question. Seventh, despite marked advances in MS instruments and techniques, the proteomics workflows need to be further simplified and standardized, and the costs need to be substantially reduced. Ultimately, identified and validated protein biomarkers should be cost-effective, provide additional information to what PSA already provides, and be easily incorporated into routine workflows in clinical laboratories.

## 9. Conclusions

The past decade has witnessed dramatic progress in MS-, antibody-, and aptamer-based global or targeted proteomics. Many promising PC protein biomarker candidates have been identified, among which some are undergoing biomarker validation in large cohorts of PC specimens. Although several hurdles must be overcome, we are optimistic that clinically valuable protein biomarkers will be identified, validated, and commercialized in the near future. In combination with other parameters including histology, clinical data, imaging, and other molecular biomarkers, protein biomarkers will guide physicians in deciding optimal personalized management for their patients.

## Figures and Tables

**Table 1:** List of candidate PC protein markers identified by MS-based discovery proteomics.

Potential Biomarker	Sample Cohort	Source	Method	Ref.
NAAA, PTK7	10 normal, 24 non-aggressive PC, 16 aggressive PC, 25 metastatic PC	Tissue (OCT)	DIA-MS (label-free N-glycoproteomics)	[53]
MSK2, CPT2, COPA, NPY	28 PC, 8 PC-adjacent normal	Tissue (FFPE)	DDA-MS (super-SILAC)	[66]
PGM3, PYCR1, GAA, HNRNPM, TALDO1, HNRNPL, GGCT, CTSH, NPEPPS, USP5, SUCLG2, HEXB, NDRG1, STEAP4, DDAH2, CTSD, COPA, TSTA3, PSMB5, TUFM, HSP90B1	14 PC, 9 matched non-malignant	Tissue (fresh frozen)	DDA-MS (label-free)	[67]
RPL28, RBM4, RPL5, NCL, ATP5H, THRAP3, H1FX, SNRPA1, RPL23, PPIB, TPD52, HNRNPL, HNRNPUL1, RALY, RPL10A, APEH, GOT1, USP14, RAB3D, DCXR, DPT, PPL, QDPR, SOD3, OLFML3, EPHX2, EMILIN1, FMOD, GDF15	4 GS3 + 3 PC, 4 GS4 + 4 PC	Tissue (frozen)	DDA-MS (label-free)	[68]
ATR, MRE11, RAD21, RAD23A, RAD23B, RAD50, RAD9A, CHEK1, XRCC5, XRCC6	12 BPH, 18 PC	Tissue (OCT)	DIA-MS (label-free)	[69]
ACO2, CS, FH, IDH3A, MDH2, OGDH, SUCLA2, SUCLG1	10 BPH, 17 untreated PC, 11 CRPC	Tissue (fresh frozen)	DIA-MS (label-free)	[70]
NDRG3, PARP1, ABHD11, SSH3	5 PC w/o metastasis, 5 PC w/lymph node metastases, 5 lymph node metastases	Tissue (FFPE)	DDA-MS (label-free)	[71]
IGKV3D-20, RNASET2, TACC2, ANXA7, LMOD1, PRCP, GYG1, NDUFV1, H1FX, APOBEC3C, CTSZ	5 BPH, 50 PC	Tissue (fresh)	DDA-MS (label-free)	[72]
TGM2, NDRG3, KLK3/PSA, AKT1, PTEN, NKX3–1, KRAS, ATM	76 PC	Tissue (OCT ^1^)	DDA-MS (label-free)	[73]
CARS2, NFKB2, ENPP4, PDSS2 (high-grade vs. low-grade); YBX1, SETSIP, FASN, PYCR1, PDSS2, FOLH1, SPON2 (high grade vs. normal); NSUN2, HEXB, HEXA, EPCAM, PYCR1 (low grade vs. normal)	9 adjacent normal, 9 low-grade PC, 9 high-grade PC	Tissue (OCT)	DDA-MS (TMT)	[64]
SRM, NOLC1, PTGIS	10 non-malignant, 8 PC, 2 metastatic	Tissue (frozen)	DDA-MS (TMT)	[74]
FASN, TPP1, SPON2	9 BPH, 8PC	Tissue	DIA-MS (label-free)	[75]
ALB, ACTG2, FLNA, MYH11, DES, TAGLN, COL6A3, HBB, ACTB, HIST1H2AH	5 BPH, 17 PC	Tissue (fresh frozen)	DDA-MS (label-free)	[76]
SFN, MME, PARK7, TIMP1, TGM4	8 extracapsular, 8 organ-confined	Direct-EPS	DDA-MS (label-free)	[77]
KLK3/PSA, PAP, MSMB, FOLH1/PSMA, TMPRSS2	6 BPH, 5PC	EPS-urine	DDA-MS (label-free)	[78]
ACPP, ATRN, GP2, KLK11, PTPRN2, NPTN, CPE, RNASE2 (low in aggressive PC). CD97, ORM1, AFM, UMOD, PTGDS, GRN, SERPINA1, CLU, LRG1, LOX, DSC2 (high in aggressive PC)	74 aggressive PC, 68 non-aggressive PC	EPS-urine	DIA-MS (label-free N-glycoproteomics)	[54]
KLK3/PSA, ACPP, TGM4, FOLH1/PSMA	12 noncancer, 12 PC	EPS urinary EV	DDA-MS (label-free)	[79]
SCIN, AMBP, FABP5, CHMP4C, CHMP2B, BAIAP2, GRN, SYTL2, CALR, CHMP4A, DNPH1	11 negative biopsy, 18 PC including 5 GS6, 7 GS 7, and 6 GS 8–9	EPS urinary EV	DDA-MS (iTRAQ ^2^)	[80]
KLK2, KLK3/PSA, FOLH1/PSMA, MSMB, ACPP, TGM4, NDRG1, NKX3-1, FKBP5, FAM129A, RAB27A, FASN, NEFH	12 BPH, 12 PC	EPS urinary EV	DDA-MS (label-free)	[81]
B2M, PGA3, MUC3	83 BPH, 90 PC	Urine	DDA-MS (iTRAQ)	[82]
TM256/C17orf61, LAMPTOR1, VATL, ADIRF, KLK3/PSA, FOLH1/PSMA, TGM4, TMPRSS2, GOLPH3	15 noncancer, 17 PC	Urinary EV	DDA-MS (label-free)	[83]
C1QB, APOA4, CO9, ANT3, VTDB, PLMN, GPX3, ITIH4, CFAI, APOH, VTNC, IBP3, CLUS, APOA2, PEDF, TETN, CD14, LG3BP, CFAH, FCN3, HPT, CO3, APOA1, APOC3, SAMP, HEMO, CO6, KLK3/PSA, A2MG, A1At, APOE, A2Gl, TTHY, C1S, ZAG, AMBP, KNG1, CO4A, AACT, CAV1, TRFE	3 PC with BCR, 3 control	Immunodepleted serum	DDA-MS (label-free)	[84]

^1^ Optimal cutting temperature. ^2^ Isobaric tag for relative and absolute quantification.

**Table 2:** List of MS-based targeted proteomics validation studies of candidate PC protein markers.

Potential Biomarker	Sample Cohort	Source	Method	Ref.
FASN, TPP1, SPON2	16 BPH, 57 PC	Tissue	PRM-MS analysis of 6 peptides corresponding to 3 target proteins	[75]
ADSV, TGM4, CD63, GLPK5, SPHMPSA, PAPP	54 noncancer, 22 low-grade PC, 31 high-grade PC	EPS urinary EV	SRM-MS analysis of 64 peptides corresponding to 64 target proteins	[109]
C1QB, APOA4, CO9, ANT3, VTDB, PLMN, GPX3, ITIH4, CFAI, APOH, VTNC, IBP3, CLUS, APOA2, PEDF, TETN, CD14, LG3BP, CFAH, FCN3, HPT, CO3, APOA1, APOC3, SAMP, HEMO, CO6, KLK3/PSA, A2MG, A1At, APOE, A2Gl, TTHY, C1S, ZAG, AMBP, KNG1, CO4A, AACT, CAV1, TRFE	86 time-point samples from 3 PC patients with BCR and 3 controls	Immunodepleted serum	SRM-MS analysis of 59 peptides corresponding to 41 target proteins	[84]
ITIH2, CD44, IGHG2, CDH13	25 aggressive PC, 25 non-aggressive PC	Serum	PRM-MS analysis of 41 N-glycosite-containing peptides corresponding to 37 target proteins	[110]

## Data Availability

Not applicable.
